# Toward precision imaging in interstitial lung disease: advances in quantitative imaging and artificial intelligence

**DOI:** 10.1097/MCP.0000000000001291

**Published:** 2026-06-17

**Authors:** Cristina Marrocchio, Michele Ligorio, Nicola Sverzellati

**Affiliations:** Unit of Radiological Sciences, University Hospital of Parma, University of Parma, Parma, Italy

**Keywords:** artificial intelligence, high resolution computed tomography, idiopathic pulmonary fibrosis, interstitial lung disease, progressive pulmonary fibrosis, quantitative imaging

## Abstract

**Purpose of review:**

To discuss the most recent developments in quantitative imaging and artificial intelligence (AI) applications in interstitial lung diseases (ILD).

**Recent findings:**

Aided by technical developments in the field, AI applications in chest imaging are increasingly being investigated, with recent algorithms showing improved performance compared with earlier techniques. This review article discusses the various roles of AI in fibrotic ILD, including diagnosis, characterization, quantification, and prognostication.

**Summary:**

Increasing evidence supports the utility of quantitative CT and AI algorithms in improving visual assessment, increasing sensitivity and inter-observer agreement, as well as providing prognostic stratification in patients with a broad range of ILD. Nevertheless, the routine clinical application of these tools remains limited.

## INTRODUCTION

Interstitial lung diseases (ILD) consist of more than 200 disorders with an overall prevalence of approximately 57.6 per 100 000 individuals [1]. The complex nature of ILD, including diverse etiologies and unpredictable disease progression, makes clinical management highly challenging. High-resolution computed tomography (HRCT) and pulmonary function tests (PFTs) represent essential tools for ILD diagnosis, assessment, and monitoring. However, they have limitations. Functional tests, including forced vital capacity (FVC) and diffusing capacity of the lungs for carbon monoxide (DLCO), provide only an indirect measure of disease extent, and are further limited by dependence on patient effort and substantial intra-individual variability [2]. Moreover, PFTs have low sensitivity to mild morphological changes and can be confounded by possible coexisting conditions such as emphysema [3^▪^]. HRCT provides a direct and detailed assessment of fibrotic abnormalities. However, its visual interpretation is subject to considerable intra- and inter-observer variability, both in pattern classification and in estimating disease extent, and low sensitivity to subtle changes between serial CT examinations [4,5].

Artificial intelligence (AI) algorithms have emerged as promising tools to overcome these limitations, providing improved reproducibility, higher sensitivity, and additional prognostic information for patient stratification [4]. These advances may allow earlier detection of progressive disease phenotypes and support more informed and personalized treatment strategies.

Over the past two decades, quantitative CT (QCT) techniques have seen significant evolution. Early approaches relied on voxel density-based histogram analysis (e.g., kurtosis, skewness, and mean density). However, these methods failed to represent the complex spatial arrangement of voxels. More recently, traditional machine learning and deep learning approaches have demonstrated markedly improved performances [2]. Deep learning models, in particular, offer high accuracy, although efforts to improve model interpretability and transparency remain important for clinical adoption.

In the context of ILD, QCT, and AI algorithms are increasingly being investigated to address key clinical needs at different stages of patient management, from diagnosis to disease monitoring and prognosis prediction. This review aims to provide an updated overview of the most recent advances in QCT and AI-based techniques for ILD assessment, exploring current and emerging applications as well as limitations and key challenges to their implementation in routine clinical practice. 

**Box 1:**
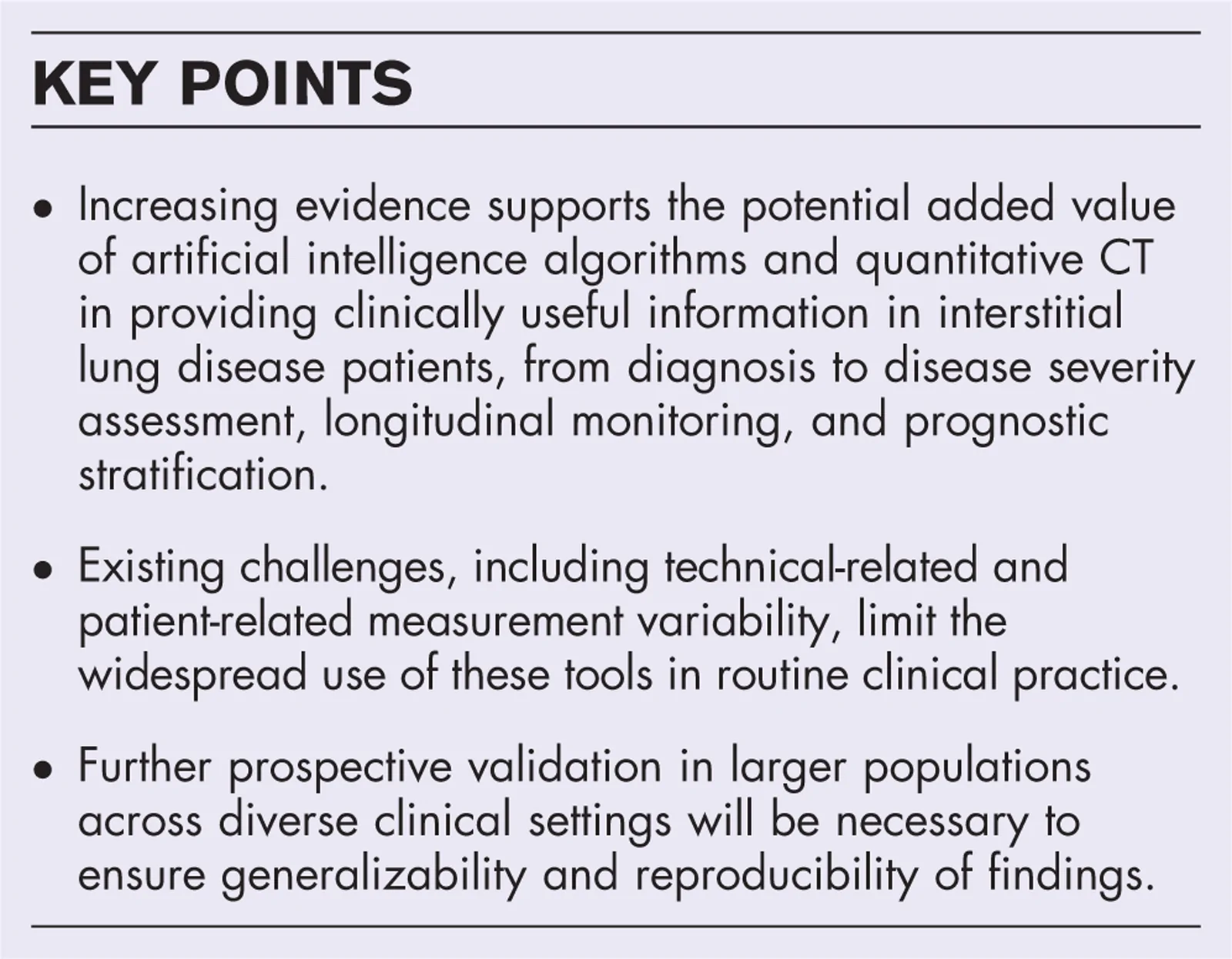
no caption available

## DISEASE DIAGNOSIS AND PHENOTYPING

While one of the main applications of AI in ILD has been the quantification of CT features, substantial research has also focused on developing algorithms to support clinicians in the complex task of ILD pattern classification, which has important diagnostic and prognostic implications.

Numerous studies have developed algorithms able to distinguish UIP from non-UIP patterns, a task of high clinical relevance for patient management. In a recent paper, Fermoyle *et al.*[6^▪^] further evaluated the clinical impact of Systematic Objective Fibrotic Imaging Analysis Algorithm (SOFIA), a deep learning model that outputs a continuous probability score (0–1) for each UIP diagnostic category [7]. A total of 312 international readers assessed 203 HRCTs from a national fibrotic registry. Readers revised their initial scores in 76% of cases after reviewing SOFIA's outputs, with increased inter-observer agreement (from moderate to good for definite UIP, from fair to moderate for the other categories). Prognostic accuracy of clinicians’ scores for transplant-free survival (TFS) and 12-month progression improved, except for the “other diagnosis” category, where performance was unchanged for progression and worsened for survival. Notably, the probable UIP category was the most revised and showed the greatest improvement in agreement and prognostic stratification [6^▪^]. SOFIA was also investigated in 522 non-idiopathic pulmonary fibrosis (IPF) patients with systemic sclerosis (SSc) [8^▪^]. After categorizing SOFIA's outputs into probability classes, no cases were classified as pathognomonic for UIP, and only 1.3% as high probability for UIP. High and intermediate probability UIP were associated with worse FVC, greater FVC decline, and worse 15-year survival, independent of clinical characteristics and baseline FVC or Goh *et al.*[9]-based staging, suggesting prognostic value independent of baseline disease severity [8^▪^].

To address the limited explainability of deep learning algorithms, Wang *et al.* developed a machine learning UIP classifier that relies on whole-lung radiomics [10^▪▪^]. In this multicenter prospective trial including 5213 HRCT, the radiomics classifier achieved an area under the curve (AUC) of 0.790 in the internal testing and 0.786 in the external validation. An integrated multimodal nomogram including radiomics, pulmonary function, and clinical variables showed improved performances (AUCs of 0.802 and 0.794 in internal and external validation, respectively) and association with higher all-cause mortality risk (HR 2.52, 95% CI 2.13–3.00) [10^▪▪^].

Humphries *et al.* described a weakly supervised deep learning model based on convolutional neural networks and multiple-instance learning (MIL), the MIL-UIP, that outperformed visual assessment in predicting histologic UIP from chest CT and provided prognostic information [11^▪▪^]. Interestingly, they showed that the highest-attention patches in their model were in the middle and upper anterior portions of the lungs, where fibrosis was mostly mild [11^▪▪^]. A more recent study also suggests that the MIL-UIP algorithm may uncover genotypic-phenotypic relationships in IPF, with multivariate analysis showing significant associations between variants in MUC5B and ZKSCAN1 and MIL-UIP scores, but not with visually assessed UIP pattern and data-driven texture analysis (DTA)-measured extent of lung fibrosis [12^▪▪^]. These findings demonstrate the potential of AI to advance our understanding of ILD, highlighting relevant imaging features and uncovering clinically significant associations with disease characteristics, including links between genetic variations and disease phenotype.

Acknowledging the need to integrate multiple domains in ILD diagnosis, Lami *et al.* developed a multimodal AI algorithm to improve UIP classification by combining chest CT and histopathological images [13^▪^]. Building on their previous pathology-based model [14], they integrated the CT imaging counterpart from a cohort of 324 ILD patients to develop a combined classifier. The model achieved an AUC of 0.92 for distinguishing UIP from non-UIP patterns and improved inter-observer agreement among pathologists in these diagnostically challenging, biopsy-proven cases [13^▪^].

Some Authors also developed algorithms to recognize other fibrotic patterns beyond UIP [15^▪▪^,^16^–18^▪^]. Of note, Wang *et al.* developed a deep learning model that utilizes MIL, showing high performance for multiclass classification in a large prospective cohort including hypersensitivity pneumonitis (HP), idiopathic non-specific interstitial pneumonia (NSIP), idiopathic cryptogenic organizing pneumonia (COP), and IPF (AUCs of 0.905 and 0.870 in two separate external validation cohorts) [15^▪▪^].

## DISEASE QUANTIFICATION AND PROGNOSTICATION

**FIGURE 1:**
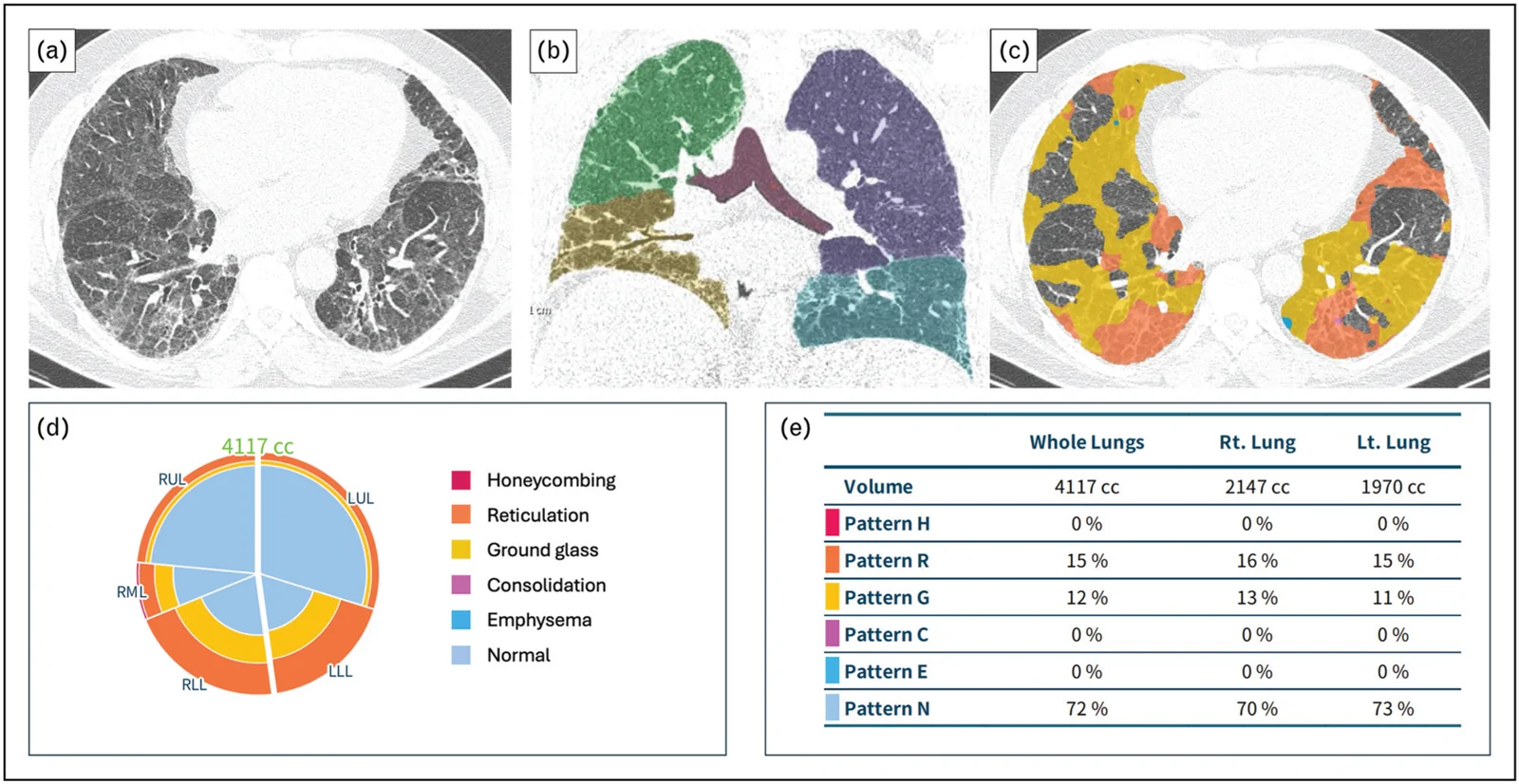
AI quantification of lung fibrosis on a chest CT scan. (a) HRCT of a patient with hypersensitivity pneumonitis, showing a bronchiolocentric interstitial pneumonia (BIP) pattern. (b) Coronal view of automated lung lobe segmentation. (c) Color overlay of automated quantification of different interstitial abnormalities. Quantitative results are shown graphically in (d), and percentages are reported for the whole lung and separately for the right and left lungs (e).

**FIGURE 2:**
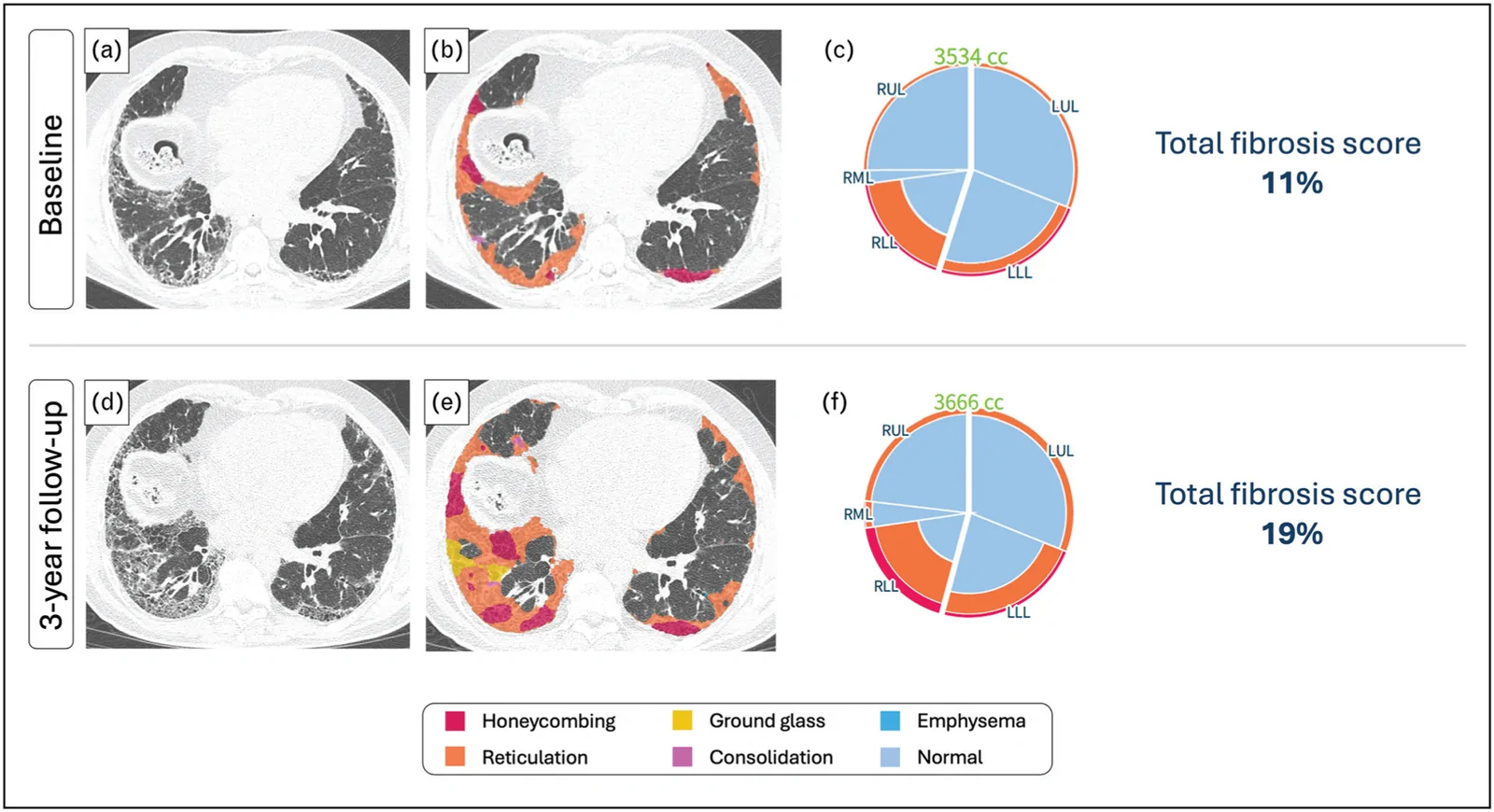
Longitudinal assessment of interstitial lung disease. HRCT of a patient with usual interstitial pneumonia (UIP) pattern at baseline (a) and at 3-year follow-up (d). Color overlays from automated quantitative analysis are shown in (b) and (e). Graphical representations of quantitative measurements (c, f) clearly show increased abnormalities, particularly in the lower lobes. The total fibrotic score, calculated as the sum of honeycombing and reticulation, increased from 11% at baseline to 19% at follow-up. The color legend is shown at the bottom.

Increasing evidence supports the role of QCT in assessing disease severity at baseline and in evaluating progression at follow-up **(**Figs. 1 and 2**)**. A systematic review summarized the evidence on automated systems for ILD monitoring and prognostication [19^▪▪^]. Among 185 included studies, results supported their efficacy across various ILD subtypes, although most data were retrospective, and a meta-analysis was not feasible due to methodological heterogeneity and lack of standardization [19^▪▪^].

To support the implementation of QCT in clinical practice, thresholds for longitudinal changes in QCT anchored to clinically meaningful outcomes, rather than relying solely on statistical significance, are being investigated. Kim *et al.* conducted a post-hoc analysis of two phase II IPF trials to determine the minimum clinically important difference (MCID) of the quantitative fibrosis score (QFS), using the percent-predicted forced vital capacity (ppFVC) and the St. George's Respiratory Questionnaire (SGRQ) as anchors [20^▪▪^]. MCIDs were 4.4% and 3.6% for a 5-point increase in SGRQ and a 3.4% reduction in ppFVC, respectively. An MCID of QLF of 1% and 2% predicted mortality, with 2% being a more conservative cut-off [20^▪▪^]. Park *et al.* investigated MCID for deep learning-derived fibrotic score changes (AVIEW, Coreline Soft, Seoul) in 476 non-IPF patients [21^▪▪^]. They reported a 1-year MCID of 2.24% and a 6-month MCID of 1.34% using an absolute FVC decline ≥5% as anchor. Changes exceeding these thresholds were independently associated with worse TFS, although the 6-month cut-off was based on a smaller effect size. QCT also provided added prognostic value in cases with discordant criteria for progression, including disagreements on visual assessment, and patients with visual-only progression showed better outcomes than patients with concordant visual-QCT progression or QCT progression alone [21^▪▪^].

Recent studies provided further evidence on the prognostic role of QCT biomarkers across a wide range of ILD subtypes [22,23^▪▪^,^24^–26^▪^]. In 407 patients with mixed ILD, 1-year changes in DTA scores predicted lung function decline and TFS, especially in patients with more limited baseline fibrosis, with results confirmed in an external validation cohort [22^▪▪^]. In another study including mixed ILD, Wang *et al.*, using a different software, found that declining total lung volumes and increasing ground-glass opacities (GGO) showed the strongest association with 2-year TFS in two independent cohorts, and derived a composite QCT measure of progressive pulmonary fibrosis (qctPPF) that was associated with increased risk of death or transplant [23^▪▪^]. Notably, agreement between visually and qctPPF-determined progression was poor, and in discordant cases, qctPPF maintained prognostic significance [23^▪▪^].

Disease-specific data are consistent with these findings. In a cohort of patients with rheumatoid arthritis-related ILD (RA-ILD), DTA scores were associated with worse baseline lung function, and both baseline and longitudinal changes were associated with risk of mortality [27^▪▪^]. In SSc-related ILD, functional parameters were correlated with deep-learning-based quantifications in a cohort of 75 SSc patients [28^▪^]. CALIPER-derived quantifications also showed correlation with functional parameters, and the baseline reticulation score was independently associated with 5-year mortality [29^▪^].

Increasing attention has also been directed to disentangling the relative prognostic impact of disease pattern and disease extent in fibrotic ILD. In a cohort of 979 patients with mixed ILD, QCT-derived fibrosis extent strongly predicted functional decline and survival, independent of visually assessed CT patterns [30^▪^]. Authors suggested that survival differences observed within and across UIP diagnostic categories were largely driven by variations in fibrosis extent [30^▪^]. In a subsequent study, Humphries *et al.* applied the MIL-UIP classifier and DTA to the same cohorts [11^▪▪^]. They suggested that the prognostic contribution of a UIP pattern decreases as fibrosis extent increases, with a UIP pattern being more informative earlier in the disease course, whereas extensive fibrosis contributes significant risk independently of pattern [11^▪▪^]. Additional evidence from two independent multicentric cohorts showed that HP with a UIP pattern had functional decline comparable to IPF patients, but CTD-ILDs with a UIP pattern had slower decline and longer TFS compared to IPF, differences that persisted when using DTA to quantify fibrosis extent [31^▪▪^]. These findings suggest that while the UIP pattern has prognostic impact, some heterogeneity may still be present according to the ILD type. However, in a recent study of non-IPF PPF patients from the ILD-PRO registry, no significant association was found between UIP pattern and disease severity or progression [32^▪▪^]. Although higher QFS was associated with severity and progression in unadjusted analyses, suggesting a stronger prognostic role than pattern, this association lost significance after adjustment for common clinical variables, indicating limited added prognostic value [32^▪▪^]. Overall, these findings highlight the complexity of isolating the independent prognostic contribution of ILD-related features, and future studies are needed to clarify their relative roles.

The potential of QCT in guiding treatment decisions and assessing therapeutic response in ILD has also been explored. In a post-hoc analysis, Matson *et al.* used machine learning to analyze HRCTs from two SSc cohorts of the SLS I and SLS II trials, and DTA to analyze HRCTs from a retrospective multicenter observational study including RA-ILD patients [33^▪▪^]. A greater extent of GGO relative to fibrosis was not associated with a physiological response to immunomodulation; however, SSc-ILD patients with higher GGO and fibrosis extent had improved response to cyclophosphamide [33^▪▪^]. A short report also presented the results from an exploratory endpoint of the TRAIL 1 trial, namely the effect of pirfenidone on QCT changes at 52 weeks in patients with RA-ILD [34^▪^]. In the 75 included subjects, DTA scores were higher in the placebo group compared to the treatment arm, even after multivariable adjustment, with a more pronounced effect in patients with a UIP pattern [34^▪^].

## NOVEL BIOMARKERS

Over the last few years, AI has allowed the extraction of novel parameters from HRCTs, beyond what can be visually assessed by radiologists. These emerging biomarkers have shown promise in providing additional prognostic information in fibrotic ILD.

### Vascular parameters

**FIGURE 3:**
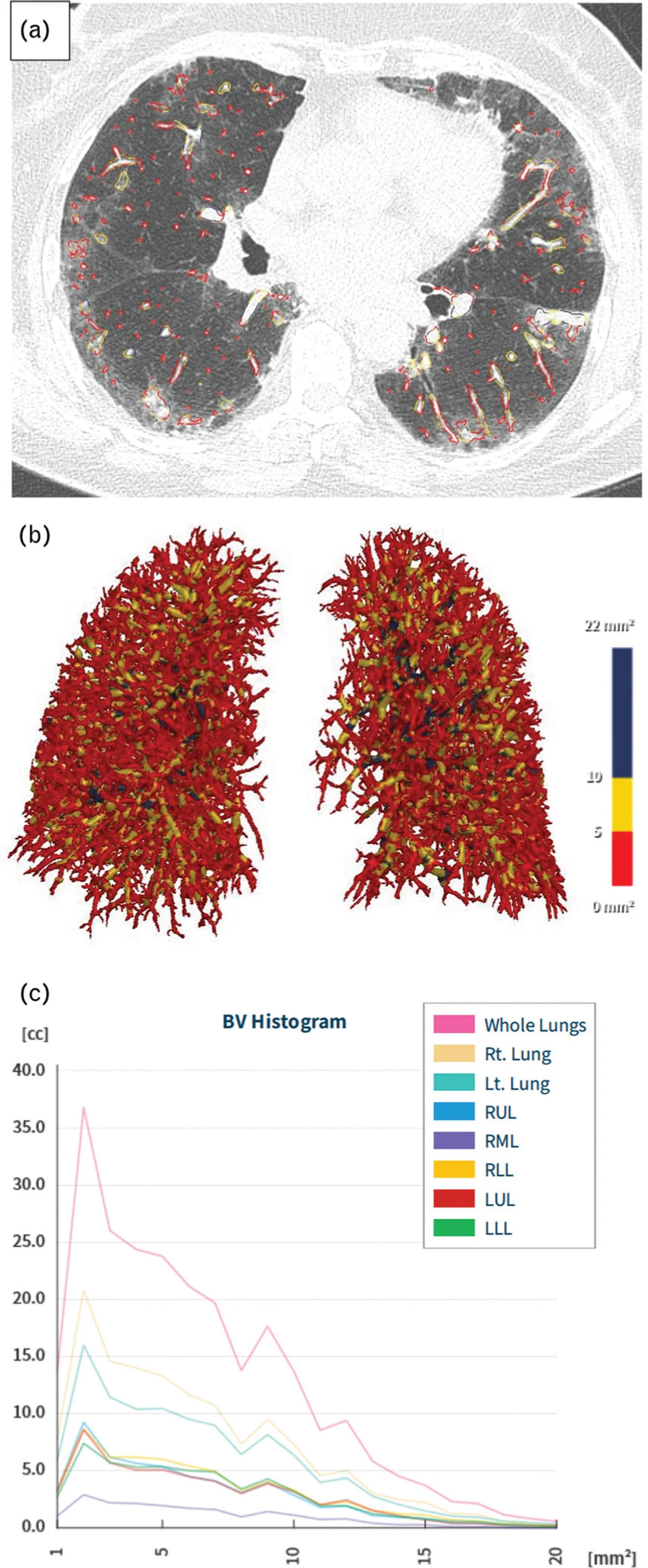
Automated lung vessels analysis. (a) Automated pulmonary vessel segmentation in a patient with systemic sclerosis-related interstitial lung disease, with color-coded differentiation of pulmonary vessels according to the vessel area (mm^2^). (b) Three-dimensional reconstruction of automated pulmonary vessel segmentation, with color coding based on vessel area (mm^2^, color legend on the right). (c) Histogram of blood volume (BV) in the whole lung and different lung regions.

Among various biomarkers, quantitative analysis of pulmonary vessels has been one of the most studied. Recent deep learning models perform rapid automated segmentation of the pulmonary vascular tree, extracting multiple morphological features such as total blood volume and the number and caliber of large and small vessels (Fig. 3). Previous studies suggested that vessel-related parameters may have a strong prognostic role in fibrotic ILD [35]. In a recent study, Li *et al.*[36^▪^] reported that the volume of small vessels (<5 mm^2^ cross-sectional area) decreased gradually with increasing Gender–Age–Physiology (GAP) stage in CTD-ILD patients, consistent with peripheral vascular pruning. Furthermore, the BV5a/v (i.e., blood volume of pulmonary arteries/veins with a cross-sectional area <5mm2) showed the strongest correlation with total lung capacity. Disease severity also correlated with vascular tortuosity [36^▪^]. However, inconsistent findings have also been reported. In the study by Wang *et al.*, vascular-related structures did not predict TFS in their cohort of mixed ILD, highlighting the need for further investigation in this field [23^▪▪^].

A novel software quantifies a reticulovascular score (RVS), which is the sum of branching vessel-like regions and linear structures with vessel-like attenuation, and a weighted RVS (WRVS), which emphasizes peripheral lung regions. In two separate IPF cohorts, a threshold of WRVS ≥15% at baseline was strongly associated with TFS when adjusted for baseline FVC and age [37^▪▪^]. A WRVS increase of 3% was associated with reduced survival, independent of FVC, and outperformed visual evaluation of progressive fibrosis [37^▪▪^].

### Other biomarkers

**FIGURE 4:**
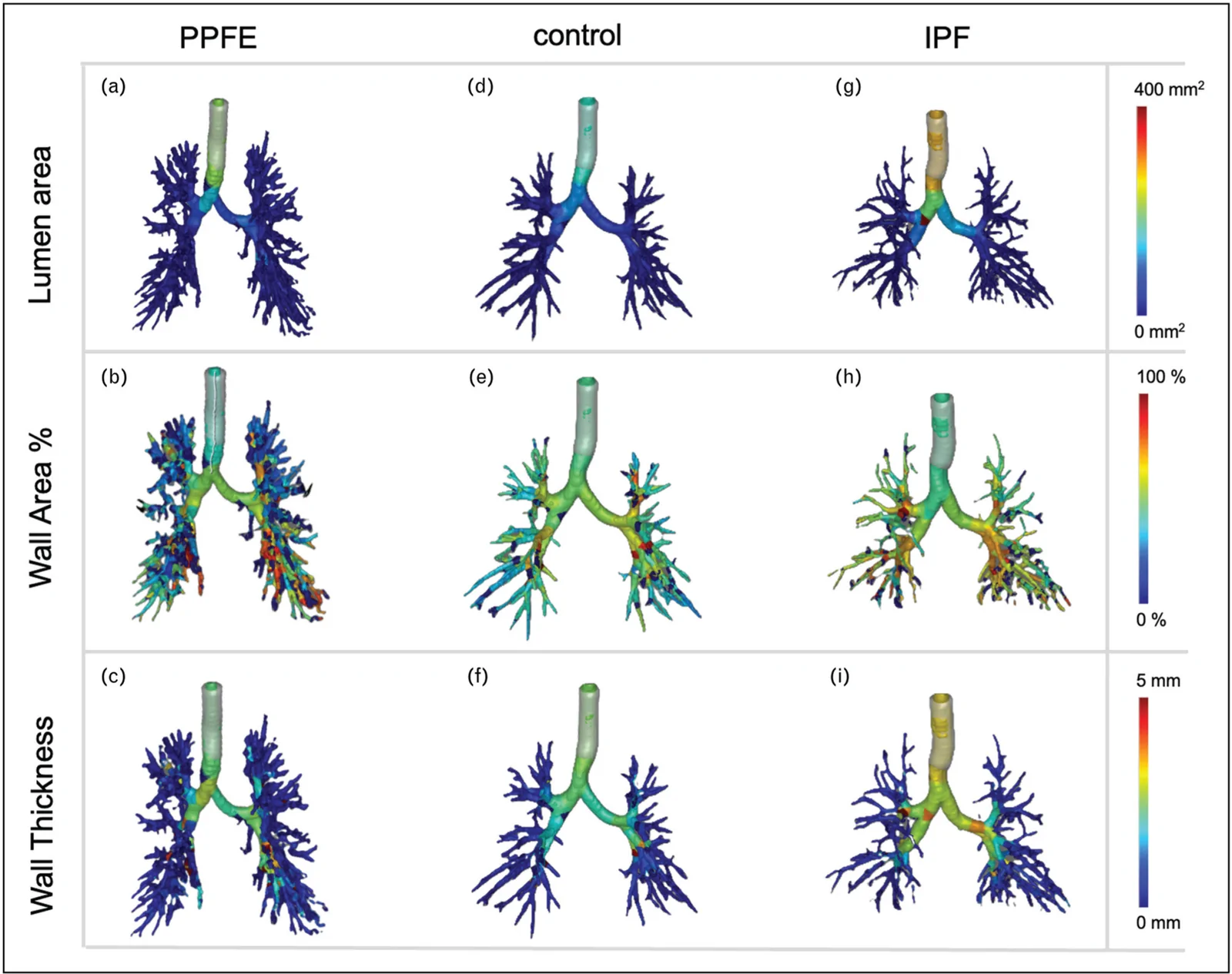
Automated airway analysis. Automated analysis of airways in three different patients with pleuroparenchymal fibroelastosis (PPFE) (a–c), no pulmonary fibrosis (d–f), and idiopathic pulmonary fibrosis (IPF) (g–i). Three-dimensional reconstructions with color-coded segmented airways are shown for three representative quantitative measures, including lumen area (a, d, g; mm^2^), wall area percentage (b, e, h; %), and wall thickness (c, f, i; mm). Corresponding color legends are shown on the right.

Evidence on novel biomarkers is still limited. While airway analysis has been widely studied in chronic obstructive pulmonary disease [38^▪^], its role in ILD remains less well defined (Fig. 4). In a prospective cohort of RA patients without a clinical diagnosis of ILD, airway wall thickness and emphysema percentages, quantified by Vision 2.2 (VIDA Diagnostics) and in-house QCT software, were associated with respiratory symptom severity, unlike PFTs and radiologist-determined airway abnormalities [39^▪^]. AirQuant algorithm calculates airway lumen diameter and length to measure segmental tortuosity and intersegmental tapering, parameters that, in a study on an IPF cohort, were proven to be associated with mortality in multivariable analyses adjusted for FVC [40]. Matsunashi *et al.*, in a cohort of 71 patients with idiopathic pleuro-parenchymal fibroelastosis (PPFE), found differences in airway metrics compared with IPF, and intrapulmonary bronchial volume fraction was independently associated with survival [41^▪^].

Extra-pulmonary biomarkers may also play a role in ILD assessment. For instance, body composition analysis has been investigated with interesting results. Lee *et al.*[42^▪^]. showed that a ≥52.3 cm^2^ decrease in fat area at T12-L1, automatically measured using deep learning, during the first year post-diagnosis, was an independent poor prognostic factor in IPF, while the change in body mass index did not show significant correlations. Salhöfer *et al.*[43^▪^]. retrospectively analyzed 79 IPF patients and showed that high fat and sarcopenia indices, alongside a low myosteatosis index, were associated with prolonged median survival and improved 5-year survival rates. Adjusted multivariate regression identified both the fat and myosteatosis indices as independent predictors of overall survival [43^▪^].

## KEY CHALLENGES TO QCT IMPLEMENTATION

Despite growing evidence supporting the added value of QCT in ILD, its use in routine clinical practice remains limited. Many challenges need to be addressed before widespread clinical adoption, ranging from establishing appropriate regulatory frameworks to conducting a comprehensive evaluation of performance across diverse clinical settings.

A major limitation is the variability of QCT measurements. Differences in CT acquisition parameters can affect automated quantifications, and clinical protocols are often heterogeneous [44]. This variability has also been shown to impact the performance of deep learning models trained to distinguish IPF from non-IPF patients [45^▪^]. To address these limitations, deep-learning mitigation strategies have been proposed. A deep learning algorithm leveraging U-Net architectures has been developed to reduce kernel-related variability [46]. A Routable Generative Adversarial Network (RouteGAN) has also been tested to reduce differences in quantifications related to several parameters (e.g., kernels, radiation dose, and different manufacturers) [47]. In the future, standardized CT protocols are highly desirable to ensure consistent and reproducible performance of AI algorithms. Patient-related factors are other potential sources of variability. Poor inspiratory efforts, motion artifacts, and concurrent acute events, such as exacerbations or infection, can affect quantifications and lead to potential misinterpretations.

Current evidence on AI in ILD is largely derived from retrospective data and post-hoc analyses. Prospective validation in larger populations, including incorporation as primary and secondary endpoints in clinical trials, is essential to ensure generalizability and reproducibility. Direct comparison with other established biomarkers, including PFTs, also requires further investigation, although some studies suggest that QCT may provide complementary information beyond functional assessment [48,49].

## CONCLUSION

AI-based analysis of HRCT in patients with fibrotic ILD shows promise for improving reproducibility of disease diagnosis and severity assessment, as well as sensitivity to longitudinal changes. Moreover, multiple AI-derived parameters have demonstrated significant prognostic value.

These findings support a potential role for AI in enabling individualized patient management, guiding tailored therapeutic decisions, and improving the identification of patients most likely to benefit from inclusion in clinical trials. However, the routine clinical implementation of these algorithms remains limited, and further studies are needed to overcome existing challenges.

## Acknowledgements

*None.*

### Financial support and sponsorship

*This work was funded by the National Plan for NRRP Complementary Investments (PNC) in the call for funding of research initiatives for technologies and innovative trajectories in the health and care sectors (PNC0000003-AdvaNced Technologies for Human-centrEd Medicine “ANTHEM”, cascade funding Spoke 3, project “Translational biomarkers to assess response to therapy in lung fibrosis – BTLF”).*

### Conflicts of interest

*There are no conflicts of interest.*

## REFERENCES AND RECOMMENDED READING

Papers of particular interest, published within the annual period of review, have been highlighted as:

▪ of special interest
▪▪ of outstanding interest
